# Upper arm length and knee height are associated with diabetes in the middle-aged and elderly: evidence from the China Health and Retirement Longitudinal Study

**DOI:** 10.1017/S1368980022001215

**Published:** 2022-05-18

**Authors:** Bingjie He, Zhengyang Li, Lu Xu, Lili Liu, Shengfeng Wang, Siyan Zhan, Yongfeng Song

**Affiliations:** 1 Department of Epidemiology and Biostatistics, School of Public Health, Peking University, 38 Xueyuan Road, Haidian District, Beijing 100191, People’s Republic of China; 2 Department of Endocrinology, Shandong Provincial Hospital Affiliated to Shandong First Medical University, Huaiyin District, Jinan, People’s Republic of China; 3 Research Center of Clinical Epidemiology, Peking University Third Hospital, Haidian District, Beijing, People’s Republic of China; 4 Center for Intelligent Public Health, Institute for Artificial Intelligence, Peking University, Beijing, People’s Republic of China; 5 Shandong Institute of Endocrine & Metabolic Diseases, Shandong First Medical University, Jinan, People’s Republic of China

**Keywords:** Diabetes incidence, Knee height, Upper arm length, Limb lengths

## Abstract

**Objective:**

To determine if limb lengths, as markers of early life environment, are associated with the risk of diabetes in China.

**Design:**

We performed a cohort analysis using data from the China Health and Retirement Longitudinal Study (CHARLS), and multivariable-adjusted Cox proportional hazard regression models were used to examine the associations between baseline limb lengths and subsequent risk of diabetes.

**Setting:**

The CHARLS, 2011–2018.

**Participants:**

The study confined the eligible subject to 10 711 adults aged over 45 years from the CHARLS.

**Results:**

During a mean follow-up period of 6·13 years, 1358 cases of incident diabetes were detected. When controlling for potential covariates, upper arm length was inversely related to diabetes (hazard ratio (HR) 0·95, 95 % CI (0·91, 0·99), *P* = 0·028), and for every 1-cm difference in knee height, the risk of diabetes decreased by about 4 % (HR 0·96, 95 % CI (0·93, 0·99), *P* = 0·023). The association between upper arm length and diabetes was only significant among females while the association between knee height and diabetes was only significant among males. In analyses stratified by BMI, significant associations between upper arm length/knee height and diabetes only existed among those who were underweight (HR 0·91, 95 % CI (0·83, 1·00), *P* = 0·049, HR 0·92, 95 % CI (0·86, 0·99), *P* = 0·031).

**Conclusions:**

Inverse associations were observed between upper arm length, knee height and the risk for diabetes development in a large Asian population, suggesting early life environment, especially infant nutritional status, may play an important role in the determination of future diabetes risk.

Diabetes mellitus is one of the largest epidemics around the world and its prevalence is increasing in both developed and developing countries, which poses a significant burden to individuals and society, especially type 2 diabetes mellitus in the middle-aged and elderly^(1–3)^. Type 2 diabetes mellitus is a complex disease and the underlying causes have not been fully elucidated. Several studies have suggested that early life environment plays a role in susceptibility to chronic disease such as CVD, hypertension, dementia and type 2 diabetes mellitus in later life^(4,5)^. Limb lengths are considered as indicators of children’s socio-economic conditions, environmental status and psychological stress, which means that differences in these anthropometric measures may reflect nutritional or other deficits throughout childhood^(6,7)^. Recently, several studies have discovered a significant association between tall stature and the lower risk of glucose intolerance and type 2 diabetes^(8–11)^.

A number of epidemiological studies have investigated the association between anthropometric parameters and risk of type 2 diabetes mellitus, while the results are inconsistent^(9–14)^. In cross-sectional analyses, some studies suggested that leg length instead of height had the strong association with diabetes^(9,10)^, and one research indicated that femur length might be the key component in this relevance^(10)^. Besides, another cross-sectional analysis found that arm lengths (including total arm length and upper arm length) were inversely related to diabetes, and forearm length, height, leg length and lower leg length were not associated with diabetes in Japanese Americans^(12)^. In prospective researches, a couple of studies also confirmed that leg length was inversely associated with the risk of developing diabetes^(11,13,14)^, but other part of limb lengths including knee height or arm length, which are also thought to be particularly sensitive to nutritional status during childhood^(15)^, has not been investigated. In addition, recent studies have found that the inverse association between limb length and diabetes was only significant in the individuals with normal fasting blood glucose or people who are lean^(16)^, and the inverse association has been observed significantly in whites, but not in African Americans^(13)^.

This association remains controversial and these studies were heterogeneous with respect to insufficient sample size (462 subjects^(14)^ or 658 participants^(12)^), study design (cross-sectional analysis), differences in outcome measurement, differences in anthropometric measures across different races and other characteristics that may contribute to inconsistencies in the literature. Previous studies mainly focused on Caucasians, Japanese Americans and African Americans, and the difference in anthropometric measures across different races suggested the need for new research in other population^(17,18)^. No prospective studies have been conducted on this topic among Asian population. Therefore, using the data from the China Health and Retirement Longitudinal Study (CHARLS) which offers a large national sample of both men and women with a relatively long period of follow-up, the present study was designed to investigate whether upper arm length and knee height are related to the risk of developing diabetes.

## Materials and methods

### Database and study population

The data used in this study came from the survey of the CHARLS. The CHARLS, a nationally representative longitudinal survey, was conducted biannually among the Chinese population aged 45 years and over, including information regarding demographics, health status and functioning, blood sample data and physical examinations^(19)^.

To date, a total of four surveys have been conducted in 2011 (visit 1), 2013 (visit 2), 2015 (visit 3) and 2018 (visit 4). Details of the design and conduct of CHARLS were described elsewhere^(19)^. The baseline survey recruited 17 424 individuals, and 13 978 (78·9 %) of them provided anthropometric and physical performance measures. For the present study, only participants who had physical examination data, did not diagnose with diabetes at baseline and had at least one follow-up were included. Individuals with missing data on upper arm length and knee height (322), with missing data on covariates (368) and with unknown diabetic status at baseline or during follow-up (661) were excluded (please note that some participants were excluded for meeting more than one criteria), accounting for 9·60 % of the population that should be included and leaving 10 711 participants included in this study.

### Measurements

Trained researchers interviewed participants face to face in their homes using computer-assisted personal interviewing technology to collect variables including socio-demographic information, health behaviour, medical history and medication usage. Physical parameters were measured by the trained investigators with standardised equipment at every follow up, and venous blood sample collection was done in 2011 (visit 1) and 2015 (visit 3)^(19,20)^. These blood samples were transported from all study sites to the study headquarters in Beijing where they were assayed for biochemical parameters including glucose, glycated HbA_1c_ and lipid profiles by trained research staff^(20)^.

### Exposure measurement

Anthropometric measures followed the survey protocol^(21)^. Upper arm length and knee height were measured by trained investigators using Dongfang^TM^ XTCL-I Martin rule. Upper arm length was defined as the distance between the olecranon process and the superior border of the acromion process, and knee height was defined as the distance from the sole of the foot to the anterior surface of the thigh, with the ankle and knee each flexed to a 90° angle. The quartiles of the limb length were divided according to gender-specific cut-off values^(11,16)^.

### Evaluation of diabetes

At baseline and any follow-up examination, subjects with one or more of the followings were determined as having diabetes: (1) fasting plasma glucose ≥ 126 mg/dl; (2) non-fasting plasma glucose ≥ 200 mg/dl; (3) HbA_1c_ ≥ 6·5 %; (4) self-reported prior diagnosis of diabetes by a doctor; and (5) self-reported use of antidiabetic medications^(22,23)^.

### Covariate assessment

Based on biologic plausibility or previous studies^(10,12,13,22)^, the following candidate covariates that were collected by trained researchers through questionnaires and physical measurements were examined as potential confounding factors: age (years), sex (male, female), marriage (single, married/divorced), smoking (never, current/former), drinking (never, current/former), weight (kg), BMI (kg/m^2^), waist circumference (cm), hypertension and dyslipidaemia. BMI was calculated by dividing weight (kg) by the square of height (m), and BMI could be classified into four levels (underweight: <18·5 kg/m^2^, normal: 18·5–24 kg/m^2^, overweight: 24–28 kg/m^2^, obesity: ≥28 kg/m^2^)^(24)^. Hypertension was defined by self-reported physician diagnosis of hypertension or blood pressure measurements ≥ 140/90 mmHg or use of antihypertensive agents^(25)^. Dyslipidaemia was defined by self-reported physician diagnosis of dyslipidaemia, or total cholesterol ≥ 240 mg/dl and LDL ≥ 160 mg/dl, or TAG ≥ 200 mg/dl, or HDL cholesterol < 40 mg/dl, or use of anti-dyslipidaemia agents^(26)^.

### Statistical analysis

Baseline characteristics of the participants in our study were presented as means with standard deviations for continuous variables, with differences between groups by diabetes status (with/without) calculated using *t* test, or percentages for categorical variables, with differences between groups by diabetes status calculated using *χ* ^*2*^ test. The Shapiro–Wilk test was used to assess for data normality. Diabetes incidence rates and 95 % CI were calculated as events per 1000 person-years using Poisson regression models. The relationship between upper arm length/knee height and onset of diabetes was conducted by survival analysis. The survival time was defined as the period from the date of first interview to the date of onset of diabetes, loss to follow-up or last interview in 2018, whichever came first. The Cox proportional hazards regression was used to obtain adjusted hazard ratios (HR) and 95 % CI. The proportional hazards assumption was tested by Schoenfeld residuals. Four models were considered to adjust potential covariates defined a priori. Model 1 was adjusted for age and sex. Model 2 was additionally adjusted for weight. In Model 3, adjustment further included marriage, smoking and drinking. Model 4 was additionally adjusted for hypertension and dyslipidaemia. To make our results nationally representative, a sample weight, which was calculated using an inverse probability method, was used under the correction for household and individual non-response as well as non-participation in the anthropometric measure. All the multivariable analyses were weighted in our study.

Subgroup analyses by sex (male, female), age (<60, ≥60), BMI (underweight, normal, overweight, obesity), hypertension status (without hypertension, with hypertension) or dyslipidaemia status (without dyslipidaemia, with dyslipidaemia) were performed using model 4. The interaction effect between limb length and a covariate was tested by including a two-way interaction term in the final model. We also performed sensitivity analysis to test the robustness of our results: we refit the Cox model to adjust the hazard of diabetes for the competing risk of death using the Fine–Gray model which estimated subdistribution hazard.

Stata 15.0 was used for all statistical analyses, and two-sided *P* value less than 0·05 was considered as statistically significant.

## Results

### Baseline characteristics

A total of 10 711 participants were included in this study, with 5114 (47·75 %) males and 5597 (52·25 %) females. The mean baseline age of all participants was 59·20 (sd 9·41) years. The participants were followed up for an average of 6·13 (sd 1·59) years, which resulted in 1358 cases of incident diabetes. Baseline characteristics by diabetes status are shown in Table 1. Compared with those without diabetes, participants with diabetes were older, female, more likely to be overweight and obese, and had shorter upper arm length at baseline. They were also less likely to be regular smokers and regular alcohol drinkers and more likely to had hypertension and dyslipidaemia at baseline. We excluded 1137 subjects because of unknown diabetic status or missing exposure and covariate information, and they were older, more likely to be female, divorced and underweight, less likely to be regular smokers and regular alcohol drinkers, and more likely to had hypertension and less likely to had dyslipidaemia at baseline (see online supplementary material, Supplemental Table S1).

**Table 1:** Baseline characteristics of study subjects subdivided by diabetes outcome

Characteristics	Overall, *n* 10 711		Diabetes, *n* 1358		Without diabetes, *n* 9353		*P*
	*n*	%	*n*	%	*n*	%	
Age (years)							
Mean	59·20		60·08		59·07		<0·001
sd	9·41		8·83		9·48		
Gender							
Male	5114	47·75	587	43·23	4527	48·40	<0·001
Female	5597	52·25	771	56·77	4826	51·60	
Marriage							
Married	9350	87·29	1176	86·60	8174	87·39	0·217
Divorced	1274	11·89	175	12·89	1099	11·75	
Single	87	0·81	7	0·52	80	0·86	
Smoking							
Never	6417	59·91	852	62·74	5565	59·50	0·011
Former	960	8·96	131	9·65	829	8·86	
Current	3334	31·13	375	27·61	2959	31·64	
Alcohol use							
Never	6310	58·91	841	61·93	5469	58·47	0·032
Former	622	5·81	81	5·96	541	5·78	
Current	3779	35·28	436	32·11	3343	35·74	

	Mean	sd	Mean	sd	Mean	sd	
BMI (kg/m^2^)	23·24	3·64	24·64	4·04	23·04	3·53	<0·001

	*n*	%	*n*	%	*n*	%	
<18·5	761	7·11	73	5·38	688	7·36	<0·001
18·5–23·9	5768	53·86	530	39·03	5238	56·01	
24·0–27·9	3061	28·58	486	35·79	2575	27·53	
≥28·0	1120	10·46	269	19·81	851	9·10	

	Mean	sd	Mean	sd	Mean	sd	
Weight (kg)	58·27	11·07	61·44	12·07	57·81	10·84	<0·001
Height (m)	1·58	0·09	1·577	0·08	1·582	0·09	0·043
Upper arm length (cm)	33·84	2·51	33·61	2·54	33·87	2·50	<0·001
Knee height (cm)	47·91	3·36	47·79	3·34	47·93	3·36	0·147
Waist circumference (cm)*	84·67	10·02	88·79	10·74	84·07	9·76	<0·001

	*n*	%	*n*	%	*n*	%	
Hypertension	3837	35·82	665	48·97	3172	33·91	<0·001
Dyslipidaemia	2743	25·61	441	32·47	2302	24·61	<0·001

* 182 participants had missing values for waist circumference.

### Upper arm length/knee height and risks of diabetes

During the study period (65 648·11 person-years, mean follow-up time 6·13 years), a total of 1358 cases of incident diabetes were detected and the incidence rate was 20·69 per 1000 person-years (95 % CI 19·61, 21·80). According to the different group of limb length, the incidence rates of diabetes during the follow-up were 22·25 (95 % CI 20·00, 24·68) per 1000 person-years in the first quartile (short limb length) of upper arm length, 21·30 (95 % CI 19·11, 23·66) in the second quartile, 19·62 (95 % CI 17·58, 21·83)in the third quartile and 19·72 (95 % CI 17·70, 21·92) in the fourth quartile (long limb length), with respective incidence rates of 21·07 (95 % CI 18·88, 23,43), 20·04 (95 % CI 17·97, 22·29), 19·13 (95 % CI 17·05, 21·40) and 22·35 (95 % CI 20·21, 24·65) per 1000 person-years in the first, second, third and fourth quartile of knee height (Table 2). With the increase of upper arm length, the incidence of diabetes had a downward trend (*P* = 0·062).

**Table 2:** Incidence of diabetes during the follow-up

Group	Number of cases	Person-years	Incidence per 1000 person-years	95 % CI
All study subjects	1358	65 648·11	20·69	19·61, 21·80
Upper arm length				
Q1	350	15 728·16	22·25	20·00, 24·68
Q2	340	15 963·77	21·30	19·11, 23·66
Q3	330	16 819·81	19·62	17·58, 21·83
Q4	338	17 136·36	19·72	17·70, 21·92
Knee height				
Q1	332	15 758·40	21·07	18·88, 23,43
Q2	334	16 665·27	20·04	17·97, 22·29
Q3	301	15 730·81	19·13	17·05, 21·40
Q4	391	17 493·63	22·35	20·21, 24·65

Quartiles of upper arm length: female: Q1: < 31·5 cm, Q2: 31·5–32·5 cm, Q3: 32·5–33·9 cm, Q4: ≥ 33·9 cm; male: Q1: < 34·0 cm, Q2: 34·0–35·3 cm, Q3: 35·3–36·5 cm, Q4: ≥ 36·5 cm.

Quartiles of knee height: female: Q1: < 44·7 cm, Q2: 44·7–46·4 cm, Q3: 46·4–48·0 cm, Q4: ≥ 48·0 cm; male: Q1: < 48·0 cm, Q2: 48·0–49·9 cm, Q3: 49·9–51·5 cm, Q4: ≥ 51·5 cm.

The average upper arm length of participants with diabetes was 33·61 cm (sd: 2·54), significantly shorter than those without diabetes (33·87 cm, sd 2·50; *P* < 0·001). When controlling for explanatory and confounding variables using Cox proportional hazards regression models, model 1 adjusted for age and sex, mode 2 adjusted for age, sex and weight, model 3 adjusted for age, sex, weight, marriage, smoking and drinking and model 4 adjusted for age, sex, weight, marriage, smoking, drinking, hypertension and dyslipidaemia all showed that upper arm length was inversely related to diabetes (model 4: adjusted HR per 1-cm difference 0·95, 95 % CI (0·91, 0·99); *P* = 0·028) (Table 3).

**Table 3:** HR (95 % CI) of incident diabetes for upper arm length and knee height, adjusted for possible explanatory and confounding factors

	Model 1			Model 2			Model 3			Model 4		
	HR	95 % CI	*P*	HR	95 % CI	*P*	HR	95 % CI	*P*	HR	95 % CI	*P*
Upper arm length	0·99	0·95, 1·04	0·774	0·96	0·91, 0·99	0·036	0·95	0·91, 0·99	0·027	0·95	0·91, 0·99	0·028
Knee height	1·01	0·98, 1·05	0·400	0·97	0·94, 0·99	0·038	0·96	0·93, 0·99	0·024	0·96	0·93, 0·99	0·023

HR, hazard ratio.

Model 1: adjusted for age and sex; model 2: adjusted for age, sex and weight; model 3: adjusted for age, sex, weight, marriage, smoking and drinking; model 4: adjusted for age, sex, weight, marriage, smoking, drinking, hypertension and dyslipidaemia.

The average knee height of participants with diabetes was 47·79 cm (sd 3·34), shorter than those without diabetes (47·93cm, sd 3·36), but the difference was marginal significant (*P* = 0·147). However, adjusted for all potential covariates, for every 1-cm difference in knee height, the risk of diabetes decreased by about 4 % (model 4: adjusted HR 0·96, 95 % CI (0·93, 0·99); *P* = 0·023) (Table 3).

### Subgroup analyses

**Fig. 1:**
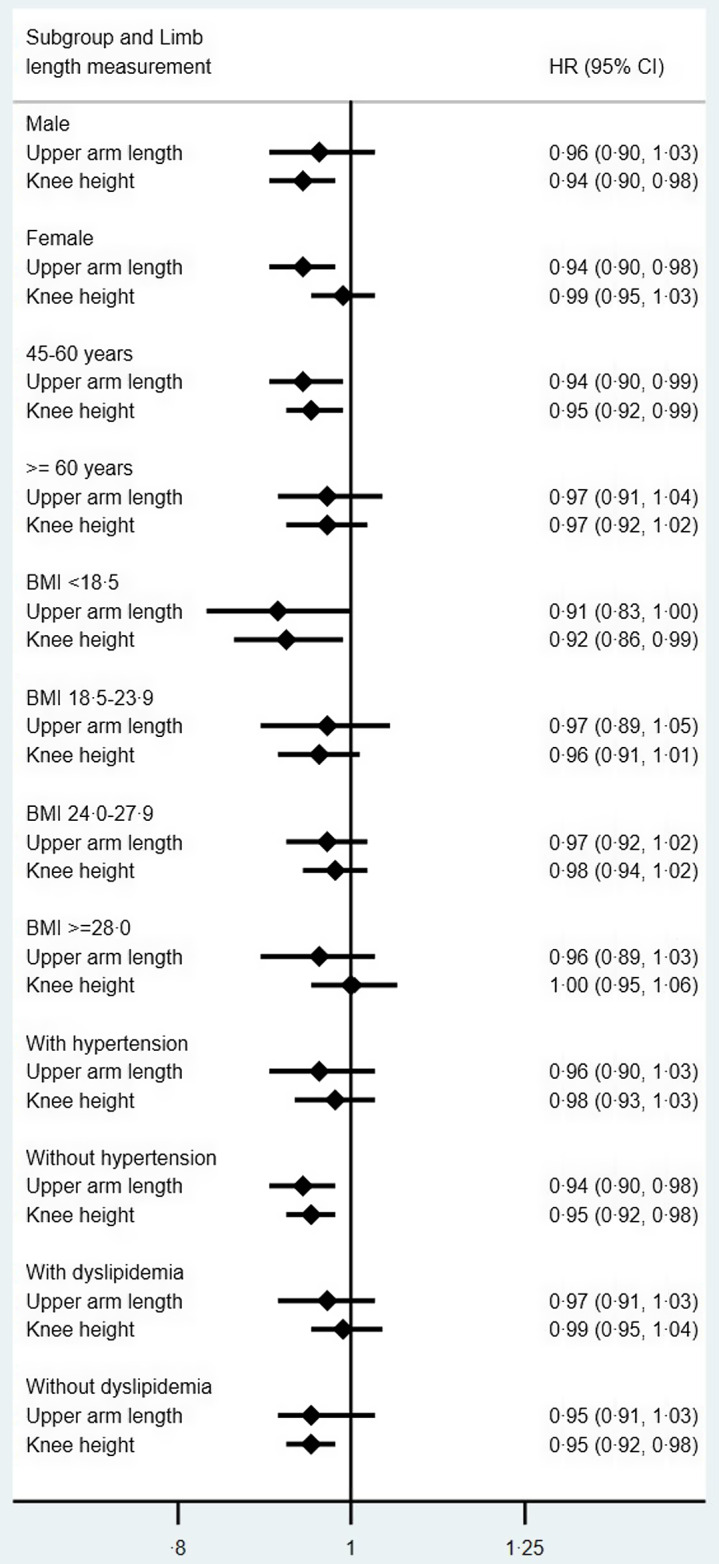
Hazard ratios (HR) and 95 % CI for the risk of diabetes, by sex, age, BMI, hypertension status or diabetes status

The results of subgroup analyses are presented in Table 4 and Fig. 1. The association between upper arm length and diabetes was only significant among females (adjusted HR 0·94, 95 % CI (0·90, 0·98)), while the association between knee height and diabetes was only significant among males (adjusted HR 0·94, 95 % CI (0·90, 0·98)), and the interaction between knee height and gender was significant (*P* = 0·042). In different age group, the associations between upper arm length/knee height and diabetes were only significant in the younger group. In analyses stratified by BMI, significant associations only existed among those who were underweight (adjusted HR 0·91, 95 % CI (0·83, 1·00); *P* = 0·049/adjusted HR 0·92, 95 % CI (0·86, 0·99); *P* = 0·031). Subgroup analyses by hypertension status and dyslipidaemia status showed that significant associations only existed among those who had no hypertension and no dyslipidaemia, and the interaction term between knee height and dyslipidaemia in the fully adjusted model was significant (*P* = 0·032) (Table 4).

**Table 4:** Association of upper arm length/knee height with development of diabetes according to subgroups of various variables

Subgroup	No. of cases	Person-years	Upper arm length			Knee height		
			HR	95 % CI	*P*_for interaction_	HR	95 % CI	*P*_for interaction_
Gender					0·302			0·042
Male	587	31 155·45	0·96	0·90, 1·03		0·94	0·90, 0·98**	
Female	771	34 492·66	0·94	0·90, 0·98**		0·99	0·95, 1·03	
Age					0·830			0·715
45–60 years	702	38 949·27	0·94	0·90, 0·99**		0·95	0·92, 0·99**	
≥60	656	26 698·84	0·97	0·91, 1·04		0·97	0·92, 1·02	
BMI					0·516			0·240
<18·5	73	4542·17	0·91	0·83, 1·00*		0·92	0·86, 0·99*	
18·5–23·9	530	35 754·15	0·97	0·89, 1·05		0·96	0·91, 1·01	
24·0–27·9	486	18 721·17	0·97	0·92, 1·02		0·98	0·94, 1·02	
≥28·0	269	6628·79	0·96	0·89, 1·03		1·00	0·95, 1·06	
Hypertension					0·701			0·225
Yes	665	22 559·64	0·96	0·90, 1·03		0·98	0·93,1·03	
No	693	43 088·47	0·94	0·90, 0·98**		0·95	0·92, 0·98**	
Dyslipidaemia					0·183			0·032
Yes	441	16 584·03	0·97	0·91, 1·03		0·99	0·95, 1·04	
No	917	49 064·07	0·95	0·91, 1·00		0·95	0·92, 0·98**	

HR, hazard ratio.

* *P* < 0·05.

** *P* < 0·01.

***P < 0·001.

Adjusted for age, sex, weight, marriage, smoking, drinking, hypertension and dyslipidaemia.

### Sensitivity analyses

The competing risk analysis did not materially alter the main results described above (upper arm length: adjusted HR per 1-cm difference 0·95, 95 % CI (0·91, 0·99), *P* = 0·029; knee height: adjusted HR per 1-cm difference 0·96, 95 % CI (0·93, 0·99), *P* = 0·017) (see online supplementary material, Supplemental Table S2).

## Discussion

In this large prospective cohort study of adults in China, inverse associations were observed between upper arm length, knee height and the risk for diabetes development, and these results were significant after rigorous adjustment. To the best of our knowledge, this is the first study to evaluate the associations of different limb lengths with the risk of diabetes development in Asia population.

Leg length was considered to be a marker for poor environmental conditions in early life^(14,27)^. Shorter leg length was found to be independently related to the risk of diabetes in white Americans^(13)^, British Women^(9)^, Canada population^(14)^ and Germany population^(11)^, but the association was not found in African Americans^(13)^, Japanese Americans^(12)^ or Chinese^(28,29)^. Another study using data from the National Health and Nutrition Examination Survey in the USA indicated that femur length might be the key height component in diabetes risk association^(10)^. Our study showed knee height as another component of leg length was significantly associated with incidence of diabetes in Chinese, but the result was faint. First, the inconsistent results of these studies on the relationship between leg length and diabetes may be due to differences in ethnicity reflected in different susceptibility to diabetes or different body composition^(30,31)^. There are racial/ethnic differences in incidence of type 2 diabetes mellitus, and these differences might be associated with many other risk factors including being obese and diet for type 2 diabetes mellitus^(30)^. Also, many studies have shown that compared with whites, Asians have different body composition^(18,32)^, and there are differences in BMI and relative leg length among Asians^(33)^. These ethnic differences might contribute to the inconsistency of the association between leg length and diabetes^(12,13)^. Second, the small sample size in the African Americans study and the Japanese Americans study could be an additional explanation for the absence of association, and homogeneity of the population and inappropriate adjustment for adult BMI in the two Chinese studies, which only recruited residents in Shanghai or Guangzhou, might explain the different results from our study. The two Chinese studies recruited permanent Guangzhou residents aged 50 years and older^(28)^ or population living in urban communities of Shanghai^(29)^ respectively, which could suggest that environmental factors in early life might be similar in two study population, and therefore, the difference in leg length or knee height would be much smaller than in a population with large variation in socio-economic status, and researchers could not found the association between leg length and diabetes. Also, the population differences in our study may not be large enough to find a greater effect. In addition, we only have data on knee height and no data on leg length, which prevents us from fully explaining the association between leg length and its components with diabetes in the Chinese population. Besides, greater knee height was associated with lower risks of dementia and Alzheimer disease in US women^(4)^, and this association was related to nutrition in early life, as is the case with type 2 diabetes as hypothesised by the thrifty phenotype hypothesis^(34)^, which could also provide evidence for knee height as a good indicator for susceptibility to chronic disease during adulthood.

We have shown that upper arm length was inversely associated with diabetes and the result was also faint after adjustment for confounding factors. Related research for arm length is relatively limited, but arm length has also been shown to be related to early life environment^(4,34)^. In a study conducted in the USA, a cross-sectional analysis of more than 600 Japanese Americans found that total arm length and upper arm length were negatively correlated with diabetes^(12)^, which is consistent with our research. Because the average upper arm length and knee height variation were faint, whether it has clinical significance remains to be studied.

The underlying mechanism for the association between limb lengths and diabetes has not been fully understood. Adult limb lengths can be affected by genetic and environmental factors, but previous studies have shown that limb lengths are associated with early childhood nutrition, especially infant nutrition^(27,34,35)^. Among the relatively undernourished populations, such as the Chinese population in our study, limb lengths may be more determined by nutritional conditions in the early life^(36–38)^. The possible explanation is the thrifty phenotype hypothesis, which believes that poor nutrition in fetal and early infant life are detrimental to the development and function of the Beta cells of the islets of Langerhans and such defects predispose to the later development of diabetes^(39–41)^. A recent experiment in rats also confirmed this view. The study found that postpartum nutritional deficiencies and subsequent catch-up growth reduced insulin sensitivity^(42)^. Our epidemiological study found that upper arm length and knee height, as indicators of environmental conditions in early childhood, were negatively correlated with diabetes, which could provide indirect evidence that poor nutritional status in early life may lead to long-term metabolic disorders and is related to the development of diabetes. As for different parts of the limb length, human beings follow a cephalo-caudal gradient of growth, which is characterised by the faster growth rate of the lower limbs, especially the knee height, than other body segments during the period from birth to puberty^(43)^. Some studies have found that different anatomical regions of the human body (humerus, femur, tibia, hand or foot) have different sensitivity to environmental pressure during growth^(44)^, and the ‘distal blood flow’ hypothesis assumes that because it is the last area to receive oxygenated blood and blood nutrients, the tibia is more susceptible to adverse factors such as hypoxia than other limbs^(45,46)^. Therefore, knee height might be more sensitive to nutrient shortage, infection and socio-economic environment in early life. This assumption still needs further verification.

In terms of subgroup analyses, the associations did have differences between genders. The association between upper arm length and diabetes was only significant among females, while the association between knee height and diabetes was only significant among males. The gender difference observed in the effect of upper arm length and knee height on diabetes risk possibly strongly associated with menarche and the menstrual cycle. In girls, increases in body fat can affect the initiation of menarche, ensuing early puberty, which influence final height in females^(47)^. When the association was separately analysed in groups divided by BMI, only those who were underweight showed significant inverse association between limb lengths and diabetes, which might indicate that the effects of obesity overwhelmed the effects of short limb lengths when individual was overweight or obese^(16,48,49)^. Several studies have revealed that early exposure to poor nutrition may also increase the risk for obesity in adulthood^(49–52)^ and obesity is an important risk factor for type 2 diabetes mellitus^(22)^, which indicates that obesity and limb lengths all reflect the nutritional status of early life to some extent and the effects of limb lengths on diabetes may be greatly affected in overweight and obese people. The competing risk analysis considering the effect of death also did not materially alter the main results, which reflected the robustness of the results of this study.

### Strengths and limitations

To our knowledge, this is the first study to investigate the association of different limb lengths with diabetes in Asian population, using nationally representative longitudinal data with relatively large sample size and an average follow-up time of 6·13 years. Nevertheless, it has some limitations. First, we only have upper arm length and knee height data and have no other limb length data, such as total arm length, arm span, leg length and femur length. Therefore, we cannot fully elucidate the association of different components of limb lengths with diabetes, but we still added the evidence of the association of limb lengths with diabetes considering the correlation between limb lengths. A second limitation is that we cannot rule out the fact that some of the subjects in our study might have type 1 diabetes. However, considering that type 2 diabetes mellitus accounts for the vast majority of diabetes patients and that the onset age of diabetes in our study population was over 45 years old, it is likely that all patients in this analysis had type 2 diabetes^(37,53)^.

## Conclusion

In conclusion, inverse associations were observed between upper arm length, knee height and the risk for diabetes development in a large Asian population. Since upper arm length and knee height may be more determined by early life nutritional conditions, our results are consistent with the hypothesis that poor nutrition in early life may lead to long-term metabolic disorders and be associated with the development of diabetes. The results of this study suggest that attention should be paid to prenatal, postpartum and early childhood nutrition to prevent adulthood diabetes, and limb length might be used as a predictor for diabetes prediction. More studies with complete limb lengths data are still needed to confirm our findings and clarify the potential underlying mechanisms.

## Supplementary material

For supplementary material accompanying this paper visit https://doi.org/10.1017/S1368980022001215. click here to view supplementary material
