# Supplementary material for: Upper arm length and knee height are associated with diabetes in the middle-aged and elderly: evidence from the China Health and Retirement Longitudinal Study
Source: Public Health Nutr. 2022 May 18;26(1):190–8. doi: 10.1017/S1368980022001215 (PMC11077445; doi:10.1017/S1368980022001215)
Supplement: Supplementary file 1 [file S1368980022001215sup.zip › S1368980022001215sup002.docx]

**Supplementary Tables**

**Table S1** Baseline characteristics of study subjects subdivided by missing status

| Characteristic | Included, n=10,711 | Excluded, n=1,137 | *P*-value |
| --- | --- | --- | --- |
| Age(yr), mean (SD) | 59.20 (9.41) | 63.33 (11.75) | <0.001 |
| Gender |  |  |  |
| Male, n(%) | 5,597(52.25) | 558(49.08) | 0.041 |
| Female, n(%) | 5,114(47.75) | 579(50.92) |  |
| Marriage |  |  |  |
| Married, n(%) | 9,350(87.29) | 887(78.08) | <0.001 |
| Divorced, n(%) | 1,274(11.89) | 226(19.89) |  |
| Single, n(%) | 87(0.81) | 23(2.02) |  |
| Smoking |  |  |  |
| Never, n(%) | 6,417(59.91) | 621(55.25) | 0.001 |
| Former, n(%) | 960(8.96) | 134(11.92) |  |
| Current, n(%) | 3,334(31.13) | 369(32.83) |  |
| Alcohol use |  |  |  |
| Never, n(%) | 6,310(58.91) | 647(58.45) | 0.005 |
| Former, n(%) | 622(5.81) | 91(8.22) |  |
| Current, n(%) | 3,779(35.28) | 369(33.33) |  |
| Body mass index (kg/m^2^), mean (SD) | 23.24(3.64) | 22.66(3.87) | <0.001 |
| <18.5, n(%) | 761(7.11) | 105(12.95) | <0.001 |
| 18.5~23.9, n(%) | 5,768(53.86) | 423(52.16) |  |
| 24.0~27.9, n(%) | 3,061(28.58) | 220(27.13) |  |
| >=28.0, n(%) | 1,120(10.46) | 63(7.77) |  |
| Weight (kg), mean (SD) | 58.27(11.07) | 56.89(12.13) | <0.001 |
| Height (m), mean (SD) | 1.58(0.09) | 1.58(0.09) | 0.453 |
| Upper arm length (cm), mean (SD) | 33.84(2.51) | 33.93(2.55) | 0.310 |
| Knee height (cm), mean (SD) | 47.91(3.36) | 47.77(3.79) | 0.235 |
| Waist circumference (cm), mean (SD) | 84.67(10.02) | 84.55(11.07) | 0.734 |
| Hypertension, n(%) | 3,837(35.82) | 496(43.89) | <0.001 |
| Dyslipidemia, n(%) | 2,743(25.61) | 218(19.53) | <0.001 |

**Table S2** Subdistribution HR (95% CI) of incident diabetes for upper arm length and knee height using Fine-Gray model, adjusted for possible explanatory and confounding factors

|  | Model 1 | | Model 2 | | Model 3 | | Model 4 | |
| --- | --- | --- | --- | --- | --- | --- | --- | --- |
|  | HR(95% CI) | *P*-value | HR(95% CI) | *P*-value | HR(95% CI) | *P*-value | HR(95% CI) | *P*-value |
| Upper arm length | 0.99(0.95,1.04) | 0.776 | 0.95(0.92,1.00) | 0.037 | 0.95(0.91,0.99) | 0.028 | 0.95(0.91,0.99) | 0.029 |
| Knee height | 1.01(0.98,1.05) | 0.451 | 0.96(0.93,1.00) | 0.029 | 0.96(0.93,0.99) | 0.018 | 0.96(0.93,0.99) | 0.017 |

Abbreviations: HR, hazard ratio.
